# Population-Level Correlates of Preterm Delivery among Black and White Women in the U.S

**DOI:** 10.1371/journal.pone.0094153

**Published:** 2014-04-16

**Authors:** Suzan L. Carmichael, Mark R. Cullen, Jonathan A. Mayo, Jeffrey B. Gould, Pooja Loftus, David K. Stevenson, Paul H. Wise, Gary M. Shaw

**Affiliations:** 1 Division of Neonatology and Developmental Medicine, Department of Pediatrics, Stanford University School of Medicine, Stanford, California, United States of America; 2 General Medical Disciplines, Stanford University School of Medicine, Stanford, California, United States of America; University of Southampton, United Kingdom

## Abstract

**Objective:**

This study examined the ability of social, demographic, environmental and health-related factors to explain geographic variability in preterm delivery among black and white women in the US and whether these factors explain black-white disparities in preterm delivery.

**Methods:**

We examined county-level prevalence of preterm delivery (20–31 or 32–36 weeks gestation) among singletons born 1998–2002. We conducted multivariable linear regression analysis to estimate the association of selected variables with preterm delivery separately for each preterm/race-ethnicity group.

**Results:**

The prevalence of preterm delivery varied two- to three-fold across U.S. counties, and the distributions were strikingly distinct for blacks and whites. Among births to blacks, regression models explained 46% of the variability in county-level risk of delivery at 20–31 weeks and 55% for delivery at 32–36 weeks (based on R-squared values). Respective percentages for whites were 67% and 71%. Models included socio-environmental/demographic and health-related variables and explained similar amounts of variability overall.

**Conclusions:**

Much of the geographic variability in preterm delivery in the US can be explained by socioeconomic, demographic and health-related characteristics of the population, but less so for blacks than whites.

## Introduction

Preterm delivery (i.e., delivery at <37 completed weeks' gestation) is one of the main causes of infant mortality in the U.S., and it is associated with substantial morbidity [1]. Its prevalence shows substantial variability geographically, for example ranging from 9–17% across U.S. states.[2] It also varies widely by race-ethnicity. Black women are twice as likely as white women to have preterm deliveries and three times more likely to have very preterm deliveries (<32 weeks), which are the most vulnerable to mortality and long-term morbidities. Many studies have tried to determine what factors explain individual-level risk of preterm delivery (e.g., [3]–[5]); fewer have focused on what explains prevalence (i.e., population-level risk).[6], [7] As Geoffrey Rose eloquently articulated several decades ago, the determinants of individual risk may not be the same as the determinants of prevalence, but both are important to understand from a prevention as well as an etiologic standpoint.[8]

Cullen et al. recently reported that most of the county-level variation in premature adult mortality (i.e., death before age 70) in the U.S. – as well as black-white disparities – was explained by 22 sociodemographic, socioeconomic, environmental, and health-related variables that were measured at the county level.[9] The study demonstrated an innovative approach to understanding geographic variability and health disparities, in that it incorporated multiple variables into a single model and focused on their combined ability to explain disparities.

Our objective here was to apply that approach to preterm delivery, given the commonalities between preterm delivery and premature adult mortality – namely that both have substantial geographic variability, black-white disparity, and are likely affected by a complex array of factors related to the social context, environment and health-related behaviors. Specifically, we examined the ability of a set of social, environmental and health-related factors to explain geographic variability in the risk of preterm delivery among live births to black and white women in the US and whether these factors explain black-white disparities in preterm delivery.

## Materials and Methods

We examined U.S. singleton births from 1998–2002, using birth certificate data from the National Center for Health Statistics final natality data. Following the methods of Cullen et al.,[9] we examined births in counties whose total population included at least 100,000 people and in US Census-defined Public Use Metadata Areas (PUMAs) for counties with <100,000 people. PUMAs represent contiguous groupings of counties, such that the resulting population includes at least 100,000 people. We used PUMAs rather than single counties to increase the stability of estimates within sparsely populated counties. For convenience, we refer to PUMAs as ‘counties’ here. There were 957 counties available for analysis; 382 were single counties, 575 were PUMAs. All analyses were conducted separately for blacks and whites. This approach follows that of Cullen et al., it avoids the assumption that associations with risk factors are the same for blacks and whites [6], [10], and it enabled us to examine models that focused on variables specific to either the black or white population, rather than some combination or average.

The outcome for analyses was prevalence or risk of preterm delivery, from 20–31 or 32–36 gestational weeks. For initial descriptive analyses, we examined prevalence of preterm delivery, defined as the number of preterm deliveries divided by the total number of deliveries with non-missing gestational age (0.7% of births in the study counties were excluded due to missing gestational age). For regression analyses, we refined the outcome to be 1) the number of deliveries at 20–31 weeks divided by that number of deliveries plus the number at 37–41 weeks or 2) the number of deliveries from 32–36 weeks divided by that number plus those from 37–41 weeks. We used this refined definition because we consider early and moderately preterm delivery as two distinct but potentially related adverse outcomes. As such, excluding one preterm group from the denominator when considering the other preterm group avoids dilution of the observed associations due to including a related outcome in the denominator. We thus refer to the refined outcome measure as ‘risk’ of preterm delivery, since it does not follow the traditional definition of prevalence. We restricted analyses to counties with at least 20 preterm deliveries during the study period (at either 20–31 weeks or 32–36 weeks), in an effort to enhance stability of the estimates. All analyses were conducted separately for black and white women.

We focused analyses on the 468 counties that had at least 20 deliveries from 20–31 weeks of gestation among black and among white women and that had complete data on all covariates, to enable comparability across models. These counties encompass 61.1% of U.S. births to white mothers (n = 6,986,984) and 90.9% of U.S. births to black mothers (n = 2,607,150) during the study period.

We conducted multivariable population-weighted ordinary least squares linear regression analysis for each preterm outcome, stratified on race-ethnicity. To adjust for differences in the size of the birth population in each county and their potential influence on model parameter estimates and variances, the weight applied to each stratified model was the total number of white or black live births in each county, respectively. Independent variables reflected a variety of exposures that may be related to reproductive health; models for black women included variables specific to black women, and white models included variables specific to white women, whenever possible. First, we included variables from Cullen et al. 's analysis of premature adult mortality, which were primarily derived from the 2000 US Census and represent county-level sociodemographics, socioeconomic level, and environmental exposures (Table 1). Variables from the Census describe black or white adults (females when appropriate) aged 30–59, age-adjusted by the direct method. In addition, we included several health-related variables derived from birth certificates, to reflect these characteristics among all black or white women giving birth in a given county during the study period.

**Table 1:** County-level study variables possibly associated with county-level prevalence of preterm delivery: Definitions and weighted means and standard deviations.*

Socioeconomic and demographic Census variables [9]:	Variable definitions	Mean (SD) Blacks	Mean (SD) Whites
Low education	Proportion of women with education <12 years	0.21 (0.07)	0.10 (0.04)
High education	Proportion of women with education >12 years	0.48 (0.10)	0.61 (0.10)
High occupation	Proportion of women with managerial or professional occupations	0.25 (0.06)	0.36 (0.06)
Income	Women's household income per adult equivalent (×10^−3^)	0.02 (0.01)	0.04 (0.01)
Poverty	Proportion of women below poverty line	0.21 (0.07)	0.07 (0.03)
Wealth (property)	Mean property value among female homeowners (×10^−6^)	0.10 (0.05)	0.17 (0.08)
Home ownership	Proportion of women who are homeowners	0.55 (0.11)	0.80 (0.08)
Wealth (property) distribution	Gini coefficient on property values (range is 0 to 1)	0.46 (0.04)	0.44 (0.03)
Between-race disparity in wealth (property)	Mean black/Mean white property values	0.60 (0.10)	0.63 (0.10)
Living without a partner	Proportion of women divorced, separated or never married	0.55 (0.06)	0.27 (0.05)
Immigrant status	Proportion of women who are not US citizens	0.04 (0.06)	0.03 (0.04)
Urban county	Metro county by census definition (yes/no)	0.85 (0.35)	0.87 (0.33)
Southern	Southern county by census definition (yes/no)	0.59 (0.49)	0.46 (0.50)
Population growth rate	Population growth rate (or shrinkage) from 1990–2000 (percent change ×10^−2^)	0.17 (0.17)	0.25 (0.18)
Percent of county population that is black	Proportion of adults self-reported as black	0.28 (0.16)	0.16 (0.12)
Black population in surrounding area	Proportion of adults in the state, excluding county, self-reported as black	0.17 (0.09)	0.15 (0.08)
**Environmental variables** [9]:			
Availability of fast food	Proportion of restaurant sales classified as from limited service establishments	0.49 (0.07)	0.48 (0.07)
Cold climate	Mean January temperature (degrees Fahrenheit ×10^−2^)	0.39 (0.12)	0.36 (0.12)
Warm climate	Mean July temperature (degrees Fahrenheit ×10^−2^)	0.78 (0.04)	0.77 (0.05)
Air pollution	Mean PM_2.5_ (fine particulate matter) concentration (mg/M^3^)	0.14 (0.02)	0.13 (0.03)
**Health-related variables from birth certificates:**			
Maternal smoking	Proportion of women reporting smoking	0.09 (0.05)	0.14 (0.06)
Maternal diabetes	Proportion of women reporting diabetes (pre-gestational or gestational)	0.03 (0.01)	0.03 (0.01)
Maternal chronic hypertension	Proportion of women reporting chronic (pre-pregnancy) hypertension	0.01 (0.01)	0.01 (0.00)
Maternal pregnancy-related hypertension	Proportion of women reporting pregnancy-related hypertension	0.04 (0.02)	0.04 (0.01)
Teen moms	Proportion of mothers <20 years old at delivery	0.20 (0.05)	0.08 (0.04)
Older moms	Proportion of mothers ≥35 years old at delivery	0.09 (0.04)	0.16 (0.06)
Late or no prenatal care	Proportion of women with late (3^rd^ trimester) or no prenatal care	0.07 (0.02)	0.02 (0.01)
Father listed	Proportion of births with father race-ethnicity and age listed	0.61 (0.12)	0.91 (0.04)

*Each study variable was derived separately for blacks and whites and restricted to women when possible; means are for the 468 counties that had at least 20 preterm deliveries at 20–31 weeks gestation.

Preliminary analyses included crime as an indicator of stress. Specifically, we examined violent crime per capita (i.e., murder and non-negligent manslaughter, forcible rape, robbery, and aggravated assault) as derived from the FBI Uniform Crime Reports (http://www.fbi.gov/about-us/cjis/ucr/ucr).[11] Crime was not significantly (p<0.05) associated with preterm delivery in any of the preliminary regression models. Given this lack of association and that a substantial number of counties were missing crime data (about 9%), we excluded this variable from further analyses.

California was the only state that did not include maternal smoking on the birth certificate during the study period. Given the importance of smoking to preliminary results, we chose to exclude California rather than exclude smoking from our primary analyses. However, given that California contributes over 10% of all US births, we conducted sensitivity analyses that included the 17 eligible California counties but excluded smoking. We also conducted sensitivity analyses that included the maximum number of counties possible for each model, rather than just the 468 common counties.

Given the relatively large number of independent variables, their inter-relatedness, and somewhat modest number of PUMAs, we were concerned about the stability and precision of the regression coefficients. We thus used forward stepwise selection to reduce the full models; specifically, with entry and stay criteria at p<0.15. Second, we conducted a principal components analysis of all of the variables and then ran regression models that included the top factors as independent variables, separately for blacks and whites.

We did the following to assess the degree to which the distributions of the independent variables explain racial-ethnic differences in preterm delivery [9]. We calculated the predicted county-level risk of preterm delivery among black women, using regression coefficients from the step-wise models for black women and inserting the corresponding values of the independent variables among black women. We then recalculated predicted risk after inserting the (‘counterfactual’) corresponding *white* values for each of the variables in the models for black women.

## Results

**Figure 1:**
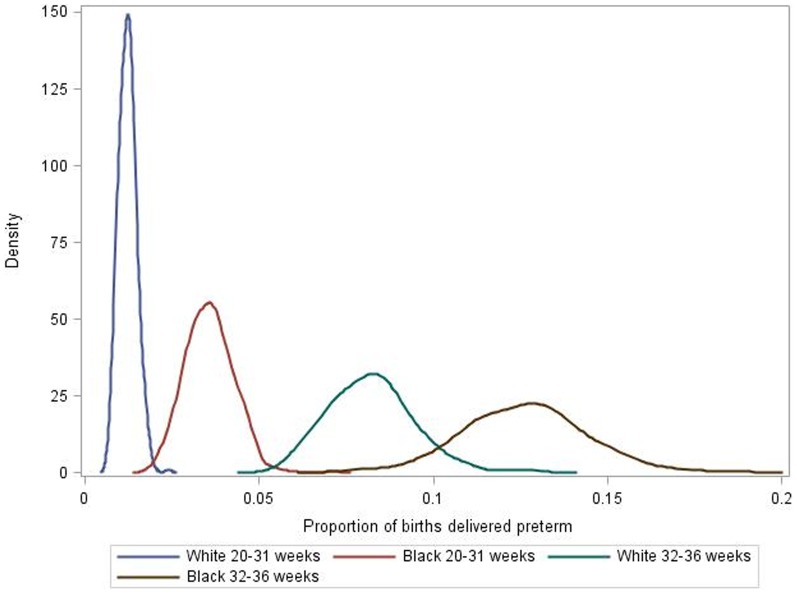
Frequency distribution (kernel plot) for the proportion of births born preterm among black and white women in 468 counties, United States, 1998–2002. * *Proportion was defined as the number of early deliveries divided by the total number of live births with non-missing gestational age. Some counties were grouped; see Methods for further detail.

The numbers of counties with at least 20 deliveries at 20–31 or 32–36 weeks and complete covariate data were 468 and 619, respectively, for black women, and 907 and 913 for white women. Figure 1 illustrates the striking difference in the distributions of preterm delivery for black and white women within the 468 counties that had at least 20 early preterm deliveries to black *and* white women. Among whites, the mean prevalence was 1.2% (range 0.7–2.4%) for delivery at 20–31 weeks and 8.2% (range 5.3–13.2%) for delivery at 32–36 weeks. Among blacks, the respective prevalences were 3.6% (range 1.9–7.1%) and 12.6% (range 7.4–18.7%).

Among births to black women, the stepwise reduced models explained 46% of the variability in county-level risk of delivery at 20–31 weeks and 55% for delivery at 32–36 weeks (based on model R-squared values). The respective percentages for whites were 67% and 71% (Table 2). Percentages were similar for the full models that included all variables and for models that included all counties with at least 20 preterm deliveries (depending on the particular analysis) rather than just the 468 counties. We also examined models that only contained the census variables, the environmental variables, or the birth certificate variables (see Table 1). Models that only included census or birth certificate variables explained similar percentages of variability in preterm delivery (32–64% for census variables, 33–62% for birth certificate variables) (Table 2). Models that only included the environmental exposure variables explained considerably less of the variability (2–38%), but these models were based on the fewest variables. Models that only included the factors from the principal components analysis explained somewhat less variability than models that included the actual independent variables. For all the models that were tested, the amount of variability explained consistently went from lowest to highest in this order: deliveries to black women at 20–31 weeks, blacks at 32–36 weeks, whites at 20–31 weeks, and whites at 32–36 weeks.

**Table 2:** Percentage of variability in county-level risk of preterm delivery explained by regression models that include varied combinations of variables or sets of counties.*

Model	Blacks, 20–31 weeks	Blacks, 32–36 weeks	Whites, 20–31 weeks	Whites, 32–36 weeks
				
Stepwise models (n = 468 counties)**	46.3	55.0	66.5	71.4
Models that included all variables or selected sets of variables (n = 468)				
All 28 variables	48.0	55.8	67.8	71.9
17 Census variables	32.1	41.9	61.5	64.0
4 Environmental variables	1.9	11.9	18.0	37.5
7 Birth certificate variables	32.9	44.4	59.8	62.0
Models including maximum number of counties with at least 20 preterm births	48.0 (n = 468)	54.4 (n = 619)	65.5 (n = 907)	69.5 (n = 913)
Models including factors from principal components analysis instead of specific independent variables (n = 468)	31.8	44.3	61.2	66.3

*Percentages are based on R-squared values from the regression models. All models include 468 common counties unless indicated as otherwise.

**Stepwise models were created using forward stepwise selection; see Table 1 for a list of variables included in each model.

A variety of variables were retained in the final regression models (Table 3). Three variables were not retained in any of the four gestation/race-ethnicity models: high occupation, availability of fast food, and air pollution. Three variables were retained in all four models: warm climate, smoking and late/no prenatal care. Most variable associations were in the expected directions (e.g., among whites, more women with low education was associated with higher preterm risk), although some were not (e.g., higher income was associated with higher risk of delivery at 32–36 weeks among whites). Among whites and blacks, there were many common correlates of preterm delivery at 20–31 and 32–36 weeks. Chronic hypertension was significant in both models for blacks and had one of the largest coefficients; i.e., a 1% absolute change in prevalence of hypertension among women giving birth was associated with a 0.21% absolute change in risk of delivery at 20–31 weeks and a 0.39% change in risk of delivery at 32–36 weeks. The set of variables in the final models tended to be different for blacks and whites.

**Table 3:** Regression coefficients for the association of county-level socioeconomic, demographic, environmental and health-related variables with county-level risk of preterm delivery.*

	Blacks		Whites		
	20–31 wks	32–36 wks	20–31 wks		32–36 wks
**Socioeconomic and demographic Census variables** [9]:					
Low education			0.011^c^	0.064^c^	
High education	0.011^b^			0.017	
High occupation					
Income				0.334^b^	
Poverty			−0.017^b^		
Wealth (property)		0.071^b^			
Home ownership	0.015^c^	0.037^c^			
Wealth (property) distribution	0.034^b^	0.093^b^			
Between-race disparity in (property) wealth		0.013			
Living without a partner			0.005^b^		
Immigrant status		−0.030			
Urban county		0.004			
Southern			0.001^c^	0.005^c^	
Population growth rate	−0.007^b^	−0.012^a^	−0.003^c^		
Percent of county population that is black	0.009^b^	0.014^a^			
Black population in surrounding area	0.012^b^				
**Environmental variables** [9]:					
Availability of fast food					
Cold climate	0.018^c^	0.047^c^			
Warm climate	0.020	0.114^c^	0.010^c^	0.060^c^	
Air pollution					
**Health-related variables from birth certificates:**					
Maternal smoking	0.067^c^	0.106^c^	0.027^c^	0.088^c^	
Maternal diabetes		−0.156		−0.075	
Maternal chronic hypertension	0.214^c^	0.389^a^			
Maternal pregnancy-related hypertension		0.167^b^		0.069^a^	
Teen moms	0.025^a^	0.110^c^			
Older moms			−0.007^b^	−0.091^c^	
Late or no prenatal care	0.080^c^	0.088^b^	0.022^b^	−0.073^a^	
Father listed	−0.009^b^	−0.027^c^			
**R-squared**	0.463	0.550	0.665	0.714	

*See Table 1 for variable definitions and scaling. Variables with p<0.15 were retained in final regression models. Regression coefficients can be interpreted as reflecting the percentage change (absolute) in risk of preterm delivery for a one percent or one-unit change in the independent variable.

a p<.05,

b = p<.01,

c = p<.001.

Patterns of results were generally similar when we excluded smoking but included California counties and when we included the maximum number of counties possible for each model, rather than just the 468 common counties (data not shown).

After inserting values of the independent variables among whites into the models for blacks (i.e., using the regression coefficients from the models for blacks), the mean predicted black-white difference in county-level risk of preterm delivery was 2.0% *higher* for delivery at 20–31 weeks (i.e., 3.0% versus 3.1%) and 14.9% lower for delivery at 32–36 weeks (i.e., 5.2% versus 4.5%). Thus, the hypothetical substitution of the values of the independent variables for whites into the step-wise models for blacks did not result in substantial explanation of the black-white disparity in preterm delivery. Given that maternal smoking was the only variable that seemed to have a substantially more ‘favorable’ distribution among blacks than whites, we re-did these calculations using models that excluded smoking. The substitution of white values of the variables resulted in a predicted risk of preterm delivery that was 32% lower for deliveries at 20–31 weeks (i.e., 3.0% versus 2.0%) and 48% lower for deliveries at 32–36 weeks (i.e., 5.2% versus 2.7%).

## Discussion

The prevalence of preterm delivery varied two- to three-fold across U.S. counties, and the prevalence distributions were strikingly distinct for black and white women. Together, the selected factors reflecting county-level socioeconomic, demographic and environmental exposures and health explained a substantial amount of the variability in risk of preterm delivery – close to 50% among black women and 70% among white women. However, these factors were less effective at explaining black-white disparities in preterm delivery. That is, underlying relationships in these factors appear relevant to preterm delivery in general but not to the disparity in preterm delivery between blacks and whites.

When analyzed separately, variables related to socioeconomic and demographic characteristics of the population (primarily from the U.S. census) and variables related to health from the birth certificate explained about the same amount of variability in the occurrence of preterm delivery. Associations with the majority of the birth certificate-derived variables were in the expected directions (e.g., more smoking or hypertension was associated with higher preterm birth). Some associations with socioeconomic and demographic variables were in expected directions, but several were not (results for high education, income, poverty, wealth and home ownership). In models that included only the socioeconomic and demographic variables, these associations were in the expected directions, except for home ownership (data not shown). Thus, even though we conducted stepwise selection to reduce collinearity, it may have affected results for these variables in the final models. Variables related to environmental exposures did not explain as much variability in preterm birth as the other variables, especially for blacks. Climate was the only environmental variable retained in final models. In all four analytic groups, warmer climate was associated with higher preterm delivery, which agrees with previous literature.[12]

When examining all variables together, differences in their distributions did not explain much of the black-white disparity in preterm birth. This is despite the fact that most of the variables had a less favorable distribution for black than white women (e.g., black women had more hypertension and lower socioeconomic level than white women). Smoking is an exception, being lower among black than white women. When smoking was excluded from these analyses, however, the models explained 32% of the black-white disparity in preterm birth at 20–31 weeks and 48% at 32–36 weeks. Our interpretation of these results is that if smoking were as prevalent among black women as it is among white women, the black-white disparity in preterm birth might be considerably greater than it is.

As noted above, most studies of preterm delivery have focused on individual-level risk factors rather than what factors explain population-level variability. One previous study examined racial-ethnic variability in the prevalence of preterm birth in the U.S. but was restricted to metropolitan areas and focused more on descriptive differences in preterm delivery by race-ethnicity than on multivariable modeling.[6] Another study focused on racial segregation and county-level prevalence of preterm delivery.[7]

Many of the variables we examined were included in a previous analysis of premature adult mortality [9], except the health-related variables from the birth certificate. The percentage of variability that was explained was higher, however, for premature mortality (72% and 79% among black and white females, respectively, and 86% and 79% among black and white males) than what we observed here for preterm delivery. In addition, differences in the distributions of the variables between blacks and whites explained the bulk of the black-white disparity in premature mortality, which was not true for preterm delivery.

Our study was limited in several ways. As an ecologic study, the results cannot be used to make individual-level inference, but they can be used to derive clues about what drives population-level variability in the occurrence of preterm delivery. We designed our analysis to parallel the analysis of premature adult mortality by Cullen et al. as closely as possible [9]. In the future, it would be useful to expand the analytic framework, for example to include other race-ethnicities (e.g., Hispanics, Asians), more recent data years, further refinement within very large counties (e.g., Los Angeles), more environmental exposures (e.g., air pollution), and multi-level analyses that compare area-level with individual-level results. We used data from 1998–2002 to parallel the Cullen et al. paper; it is possible that the observed associations may have changed over time. We consider early and moderately preterm delivery as two distinct but potentially related adverse outcomes. Individual-level studies that examine multiple degrees of preterm delivery typically restrict their comparison group to term deliveries (e.g., [13]). We used an analogous approach for our analysis; i.e., we excluded one preterm group from the denominator when considering the other preterm group. This was particularly important for analyses of early preterm delivery, because moderately preterm delivery is relatively common and its inclusion in the denominator could thus potentially ‘dilute’ associations with early preterm delivery. A limitation, however, is that this approach does alter the interpretation of our results somewhat, because the outcome does not translate to a traditional estimate of prevalence.

This study has illustrated that much of the geographic variability in preterm delivery can be explained by socioeconomic, demographic and health-related characteristics of the population, but less so for blacks than whites. Importantly, however, differences in the distribution of these characteristics between blacks and whites did not explain the marked black-white disparities in preterm delivery. Additional area-level studies are needed to determine what factors explain the remaining variability in prevalence of preterm delivery. Areas of inquiry that we believe are particularly important to explore further are environmental stressors, quality of health care, and more detailed indicators of racial-ethnic and socioeconomic disparity. As has been the case with individual-level risk of preterm delivery, it seems that explaining variability in its prevalence is also a complex challenge. Despite such difficulties, area-level studies provide clues that can be further investigated at the individual level, and they are also important to the development of effective population-level policies aimed at reducing preterm delivery.
